# Data Fusion in Agriculture: Resolving Ambiguities and Closing Data Gaps

**DOI:** 10.3390/s22062285

**Published:** 2022-03-16

**Authors:** Jayme Garcia Arnal Barbedo

**Affiliations:** Embrapa Digital Agriculture, Campinas 13083-886, SP, Brazil; jayme.barbedo@embrapa.br; Tel.: +55-19-3211-5880

**Keywords:** data fusion, sensors, variability, precision agriculture, artificial intelligence

## Abstract

Acquiring useful data from agricultural areas has always been somewhat of a challenge, as these are often expansive, remote, and vulnerable to weather events. Despite these challenges, as technologies evolve and prices drop, a surge of new data are being collected. Although a wealth of data are being collected at different scales (i.e., proximal, aerial, satellite, ancillary data), this has been geographically unequal, causing certain areas to be virtually devoid of useful data to help face their specific challenges. However, even in areas with available resources and good infrastructure, data and knowledge gaps are still prevalent, because agricultural environments are mostly uncontrolled and there are vast numbers of factors that need to be taken into account and properly measured for a full characterization of a given area. As a result, data from a single sensor type are frequently unable to provide unambiguous answers, even with very effective algorithms, and even if the problem at hand is well defined and limited in scope. Fusing the information contained in different sensors and in data from different types is one possible solution that has been explored for some decades. The idea behind data fusion involves exploring complementarities and synergies of different kinds of data in order to extract more reliable and useful information about the areas being analyzed. While some success has been achieved, there are still many challenges that prevent a more widespread adoption of this type of approach. This is particularly true for the highly complex environments found in agricultural areas. In this article, we provide a comprehensive overview on the data fusion applied to agricultural problems; we present the main successes, highlight the main challenges that remain, and suggest possible directions for future research.

## 1. Introduction

The number (and quality) of sensors used to collect data in different contexts have been steadily growing. Even complex environments, such as agricultural areas, are now being “sensed” via a wide variety of equipment, generating vast amounts of data that can be explored to provide useful information about the area being observed. As a result, the number of studies attempting to explore the wealth of information contained in the sensed data have increased [1,2,3]. However, it is often challenging to translate the advancements achieved in experiments to the conditions found in practice. There are two main reasons for this. First, the studies described in scientific texts are usually limited in scope, because the data used in these experiments usually do not cover all of the variabilities associated with the problem at hand. As a result, while the results reported in those articles may seem encouraging, they often reveal nothing about the performance of the proposed technique under real, unconstrained conditions. Second, even if the data adequately cover the variable conditions found in practice, the adopted sensing technology may not be capable of acquiring enough information to unambiguously resolve the data and provide enough information. For example, even powerful artificial intelligence models fed with RGB digital images are often unsuccessful in recognizing plant diseases from their symptoms, because different disorders can produce similar visual signs [4].

One way to reduce the gaps caused by data limitations is to apply data fusion techniques. The term “data fusion” can be defined as “the process of combining data from multiple sources to produce more accurate, consistent, and concise information than that provided by any individual data source” [5]. Other stricter definitions do exist to better fit narrower contexts. This type of approach has been applied to agricultural problems since the first half of the 1990s [6], and there has been an increase in the use of this approach. Arguably, the main challenge involved in the use of data fusion techniques involves finding the best approach to fully explore the synergy and complementarities that potentially exist between different types of data and data sources. This is particularly true with data having significantly disparate characteristics (for example, digital images and meteorological data).

It is difficult to find a formalization for the data fusion process that fits all agricultural applications, given the variety of data sources and approaches. The formalization presented by Bleiholder and Naumann [7], although derived in a slightly different context, adopts a three-step view of the data fusion process that is applicable in most cases. In the first step, the corresponding attributes that are used to describe the information in different sources need to be identified. Such a correspondence can be easily identified if the data sources are similar, but it can be challenging as the different types of data are being used. This is one of the main reasons for the existence of the three types of data fusion described in the following paragraph. In the second step, the different objects that are described in the data sources need to be identified and aligned. This step is particularly important when data sources are images, because misalignments can lead to inconsistent representations and, as a result, to unreliable answers. Once the data are properly identified and consistent, the actual data fusion can be applied in the third step. In practice, coping with existing data inconsistencies is often ignored [7]. This situation can be (at least partially) remedied by auxiliary tools, such as data profile techniques, which can reduce inconsistencies by extracting and exploring the metadata associated to the data being fused [8].

**Figure 1:**
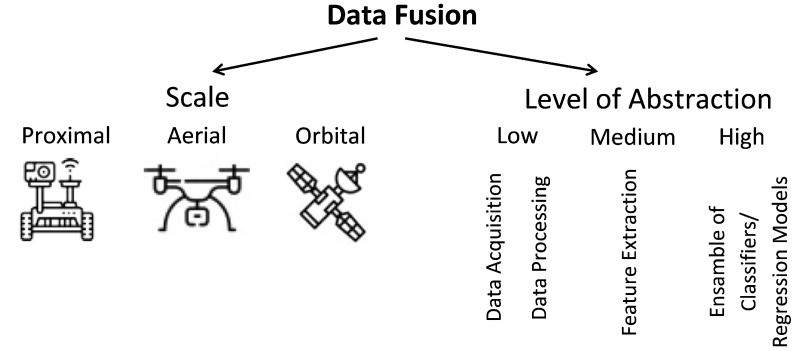
Categorization of data fusion approaches adopted in this work.

The most common categorization divides data fusion techniques into three groups [9]: (a) raw data level (also denoted “low-level” and “early integration”), in which different types of data (raw or preprocessed) are simply concatenated into a single matrix, being used in cases in which pieces of data are of the same nature and were properly normalized. (b) Feature level (also denoted “mid-level” and “intermediate integration”), in which features are first extracted from different types of data and then concatenated into a matrix, being mostly used when pieces of data can be treated in such a way they generate features that are compatible and complementary. (c) Decision level (also denoted “high level” and “late integration”), in which classification and regression algorithms are applied separately to each type of datum and then the outputs generated by each model are combined, being more appropriate when data sources are too distinct to be combined at an earlier stage. An alternative classification of data fusion methods was proposed by Ouhami et al. [10]: probability-based, evidence-based, and knowledge-based. Although both classifications are useful, the first one is more appropriate in the context of this work (Figure 1).

In the specific case of agriculture, data can be collected at three different scales—proximal, aerial, and orbital (satellites) (Figure 1). Applications that use proximal data include navigation systems for autonomous vehicles [11,12,13,14,15,16,17], fruit detection [18,19,20,21], plant disease detection [22,23,24], delineation of homogeneous management zones [25,26,27,28,29], soil analysis [30,31,32,33,34,35,36], plant phenotyping [37], among others. Aerial data (collected using UAVs) is used mostly for detection of certain objects (e.g., certain plant species and fruits) [38] and for estimation of agricultural variables (e.g., soil moisture and nitrogen content) [39,40,41]. Satellite data are used for mapping variables as diverse as soil moisture [42,43,44], crop type [45,46,47,48,49,50], crop phenological states [51,52], evapotranspiration [40,53,54,55,56,57,58], nitrogen status [59,60,61,62], biomass [63,64], among others. While most data fusion approaches only use data in the same scale, a few studies have applied data originating from different scales [10,26,28,31,38,40,51,52,64,65,66,67,68,69,70,71].

The objective of this article was to characterize the current state-of-the-art regarding the process of applying data fusion for agricultural applications. First, a comprehensive overview of the literature is provided, with emphasis on articles published after 2010. Then, the aspects involved in the use of data fusion to different types of data are explored in detail, together with some possible solutions for the weaknesses that prevent technologies based on data fusion from being more widely used in practice. Although there have been a few reviews dedicated toward data fusion in agriculture, they focused on specific applications and themes [9,10,68,70,72], while this one adopts a more general and systemic view of the subject.

The remainder of the article is organized as follows. Section 2 describes the literature related to the three different scales—proximal, aerial, and orbital. A discussion on several aspects relevant to data fusion in agriculture is presented in Section 3. Finally, Section 4 offers some final remarks and possible directions for future research.

## 2. Literature Review

**Figure 2:**
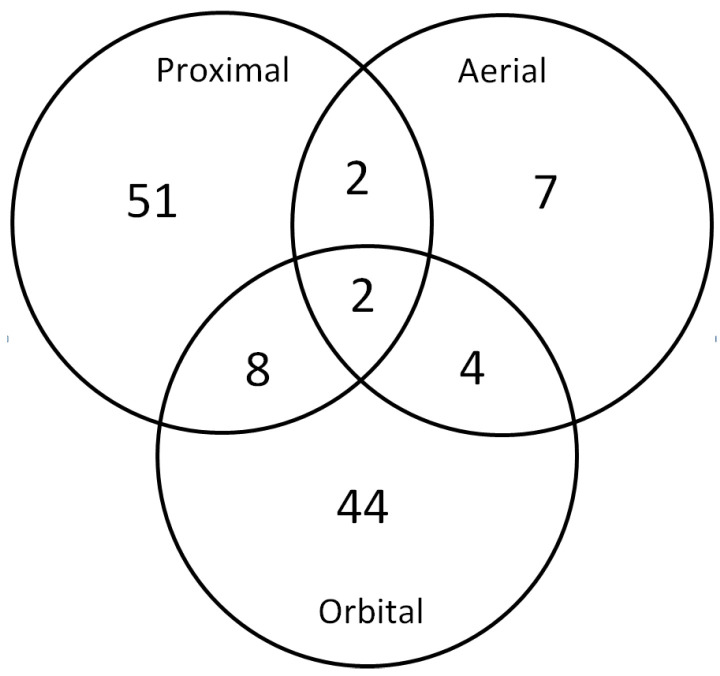
Number of references selected in this review.

A search was carried out via Google Scholar and Scopus using the keywords “data fusion” and “agriculture”. Almost 400 articles were originally selected, but this number was reduced to 119 after we removed low quality documents or documents outside of the scope of this work. From this total, 50 explore proximally collected data, 45 explore satellite data, 8 explore aerial (UAV) data, and 16 use data collected at multiple scales (Figure 2). Each scale is treated separately in this section, as they have some peculiarities that require more focused descriptions and analyses. To improve the legibility of the tables containing the full list of references, many acronyms are used, all of which are defined in Table 1.

To better organize the literature presented in this section and the subsequent discussion, both the data fusion techniques and the data being fused were categorized in a few more general classes (Table 2).

### 2.1. Proximal Scale

The majority of studies dedicated to the proximal scale are concentrated in three main areas: prediction of soil properties, delineation of homogeneous zones, and robotic navigation and control. Applications, such as disease and fruit detection, prediction of water content and water stress, estimation of phonological state and yield prediction, are also present. Ten of the references also explored satellite data, and five studies combined proximal and aerial data. Data sources included cameras (RGB, multispectral, thermal, hyperspectral) spectrometers, conductance and resistivity sensors, GPS, inertial sensors, weather data, among many others. With such a variety of sensors available for field applications, efforts to explore their complementarities have been steadily growing (Table 3), but most problems still lack reliable solutions [73].

### 2.2. Aerial Scale

Studies employing UAVs to solve agricultural problems are growing in number, but they are still outnumbered by proximal and orbital approaches. Most studies are dedicated to crop monitoring and object detection (weed, crops, etc.), although applications, such as phenotyping and water management, are also present. Almost all techniques are based on some kind of digital image (RGB, multispectral, thermal, hyperspectral). Many approaches explore the complementarity of aerial images with proximal (four articles) and orbital (six articles) data. Only eight studies employed the aerial data alone (Table 4).

### 2.3. Orbital Scale

A large portion of the articles employing satellite images aimed to either compensate for data gaps present in a primary data source by fusing it with another source of data (for example, combining optical and SAR images) [6,45,47,48,49,51,105,106], or increase the spatial resolution of the relatively coarse images collected by satellites with high revisit frequencies [42,43,44,55,57,58,107,108,109,110]. In the latter, the fused results usually inherit the details of the high spatial resolution images and the temporal revisit the frequencies of their counterparts, although the quality of the fused data usually do not match that obtained through actual missions, especially when surface changes are rapid and subtle [72]. As argued by Tao et al. [111], different sensors and image processing algorithms lead inevitably to data with some level of inconsistency, which can make rapid changes difficult to detect.

Landsat and MODIS images and products still dominate, but other satellite constellations, such as Sentinel, Worldview, GeoEye, and others, are being increasingly adopted. Data fusion has been applied to satellite images for quite some time, and well established techniques, such as STARFM and its variants, are still often used, but the interest for machine learning techniques, especially in the form of deep learning models, has been growing consistently. Water management in its several forms (evapotranspiration estimation, mapping of irrigated areas, drought detection, etc.) is by far the most common application. Yield estimation, crop monitoring, land cover classification, and prediction of soil properties are also common applications.

A major challenge associated with the orbital scale is the existence of highly heterogeneous regions with a high degree of fragmentation [109,112]. Solutions to this problem are not trivial and, as stated by Masiza et al. [113], “…successful mapping of a fragmented agricultural landscape is a function of objectively derived datasets, adapted to geographic context, and an informed optimization of mapping algorithms”. However, there are cases in which target areas can have sizes smaller than the pixel resolution of the satellite images [53]. In theses cases, pairing the images with images or other types of data obtained at higher resolutions (aerial or proximal) may be the only viable solution. Satellite data were fused together with proximal and aerial data in ten and six studies, respectively (Table 5).

Another important challenge is the difficulty of obtaining/collecting reference data for validation of the techniques applied. This problem can be particularly difficult if the reference data need to be gathered in-loco. It is also important to consider that, even if reference data can be collected, differences in granularity and the positions of the sample points can make the comparison with the fused data difficult or even unfeasible [112].

These and other challenges related to data fusion applied to satellite data were discussed in depth in [9], so they are not explored in detail here, although some of them are revisited in a more general context in the next section.

## 3. Discussion

### 3.1. Comparison of the Results Yielded by Fused and Individual Sources of Data

The last columns of Table 3, Table 4 and Table 5 show the accuracies reported in each study considered in this article. There are a few important observations to be made before analysing those results. First, the value “N/A” is used in three situations: when accuracy values are not applicable (for example in review articles), when accuracy values are not available, and when the performance of the proposed models is evaluated using either qualitative criteria or metrics that cannot be summarized in a few numbers. Second, some studies report a wide range of accuracy values. This happens when different experimental setups are adopted and also when different variables are considered. This is particularly prevalent in studies dealing with soil variables, in which case, the effectiveness of models can vary significantly from variable to variable. Third, the application of data fusion in agriculture vary significantly, so does the way accuracies are assessed, which partially explains the wide differences observed in the tables. More importantly, even the results reported in studies dealing with similar applications cannot, in general, be directly compared, unless the exact same datasets were used in the experiments. Because of these limitations, the analysis in this section focuses on the effects of data fusion, and not on differences between studies. However, it is worth noting that not all studies explicitly compare results produced with and without data fusion.

The impact of data fusion varies with the type of application. Studies that focused on the fusion of digital images invariably showed improvements with respect to the results obtained using single data sources [18,19,39,84,99,106], although in some cases the improvement may not be substantial enough to justify the capture of additional data [21,71]. The success of data fusion applied to digital images can be linked not only to the complementarities shown by different types of images, but also to the fact that the images can be easily made compatible using simple normalization operations when needed. As a result, proven techniques, such as deep learning, can be applied. In the case of agriculture, image data fusion has been particularly effective at the orbital level, both for artificially increasing the spatial resolution of sources with revisit frequencies [43,53,57,107,108,114] and for compensating cloud cover using the information present in SAR images [45,47,51,113]. In these cases, the improvement can exceed 50% [66].

The use of data fusion for the estimation of soil variables in agricultural areas is more complex, due to the significant differences between the variables that are normally considered. In these cases, data fusion can be effective at improving the estimation of some variables [30,33,35,36], but may fail to produce any improvement for others [31,33,35,95], mostly because those variables do not correlate well with any of the current soil sensors. Some studies also remark that no single data fusion approach works for all soil variables of interest, so a comprehensive variable estimation may require the use of multiple techniques [35,92].

Conversely, the usefulness of data fusion for determining homogeneous zones and producing soil maps, which usually employ soil property measurements, can also be highly dependent on the sensed data, as the inclusion of certain variables can actually decrease accuracy due to the weak link with the properties being used to determine homogeneous areas [90]. This is not a trivial task, and although some studies report encouraging results [28,95], in many cases, further research is needed to better understand how to properly explore the data collected by the sensors [25,27,76]. This has led some authors to remark that the choice of data is more important than the data fusion method employed [90].

Another application in which the use of data fusion has some particular characteristics is the positioning and navigation of autonomous vehicles to be deployed in agricultural areas. In all studies considered in this article, fusing different sensors led to decreased errors, as long as the data fusion methods were calibrated correctly [11,12,13,14,16,17,98]. It is worth noting that a variety of different vehicles were considered in those studies, and the error requirements varied from case-to-case. As a result, the level of success of the data fusion process is relative; that is, the same error levels could be considered a success or a failure depending on the vehicle and environment where it will be used.

Some of the studies considered in this article use types of data that are more particular and do not fit a general category, normally in combination with some of the more commonly used variables [24,44,64,67,93]. In general, the performance of the data fusion reported on in those cases compares favorably with single data sources, although some difficulties related with data compatibility have also been reported [82,129].

### 3.2. Data Fusion Techniques

The variety of data fusion approaches found in the literature indicates that there is no technique that fits all (or even the majority) of the possible applications. Tavares et al. [92] argued that the best data fusion approach depends on the application and attributes considered, and that the selection of an appropriate method should be conducted using independent sample subsets (independent validations) to avoid bias. It is worth noting that, depending on the problem being addressed, experiments sometimes reveal that data fusion is simply not effective [41]. It is important to keep in mind that data fusion is not always the best approach to prevent the practice of attempting to force the results to fit the original hypothesis, which often leads to biased and unrealistic claims.

The use of conventional regression models (proximal and aerial scales) and well-established spatiotemporal methods (orbital scale) is still prevalent. These types of methods have been exhaustively tested and have consistently yielded good results. Because different studies usually employ different datasets, it is not possible to make a direct comparison, but the general trend seems to indicate a lack of progress and a certain level of redundancy between studies using this type of approach. Machine learning techniques have also been applied for some time, but their full potential has yet to be realized. There are a few reasons for this. First, practicing data fusion using machine learning and artificial intelligence models is far from straightforward. Different types of data need to be handled properly in order to make them compatible within the confines of the model. Since many machine learning techniques have poor interpretability [83], it is often difficult to determine how to achieve an acceptable degree of compatibility. Second, there are a lack of databases that properly cover the entire variability associated to a given application. Although this is a problem for any type of technique [48], the effects of data gaps become more evident when machine learning techniques are applied. Third, machine learning techniques (deep learning in particular) require large amounts of computational power for model training. This problem has become much less damaging in recent years as the computational power availability increases [1]. This, combined with the fact that deep leaning has shown remarkable potential for combining and extracting information from different types of images [21,45,81], will probably lead to this type of technique being increasingly used in the future.

### 3.3. Data Fusion Level

Even the level of fusion to be adopted is not a straightforward choice. The majority of studies employ low-level fusion, arguably because this is a more straightforward and computationally lighter approach [121]. Some studies seem to indicate that higher fusion levels tend to produce better results [30,77,80], arguing that the poorer results observed when lower level fusion is applied is likely the result of data redundancies arising from complementary information from distinct sensors [30]. Other studies have come to the opposite conclusion, with results favoring the low-level approach [113]. Because few studies have compared the different fusion levels, no definitive conclusions on the matter can be drawn. More comparative studies tackling different fusion levels are needed for a better understanding on how to treat data under different contexts and conditions.

Low-level fusion attempts to directly combine different types of data directly. Since these have different value ranges and formats, some kind of normalization is almost always needed in order to make those different databases compatible [77]. If the only difference is the range of values, normalization tends to be relatively straightforward. On the other hand, if the types of data employed are of different natures (for example, images and meteorological data), the normalization process can be complex or even unfeasible, in which case, the use of higher data fusion levels may be inevitable.

Mid-level fusion can be viewed as a two-step information extraction procedure, as data are first processed in order to generate meaningful features, which are then combined into the final answer. Although in most cases the features are extracted from different types of data (the features themselves are often also different), in some cases, different features are extracted from the same dataset [79]. Calling the combination of features originating from the same dataset “data fusion” is not appropriate in most cases, but sometimes those features represent such different aspects of the data, where the techniques used to combine them are akin those employed in actual data fusion.

As mentioned before, high-level fusion tends to yield solid results, but this approach is the least used among the three levels of fusion. The explanation for this seems to be related to two main factors. First, high-level fusion can be computationally expensive, especially during training [121]. This is particularly true if one or more of the classifiers are based on deep neural networks. Second, choosing the right variables to feed a single model usually is challenging enough, so the process of selecting variables for multiple models may become somewhat impractical. Nevertheless, the use of high-level fusion tends to grow as the availability of computational resources becomes less of a problem, as the research on data fusion matures, and as the characteristics of each type of model/classifier become better understood.

### 3.4. Differences between Fusion Techniques

Directly comparing the results yielded by different data fusion techniques is difficult for several reasons: the datasets used in the studies are different in terms of data distribution and characteristics of the experimental areas, assessment criteria are diverse, and constraints on the experimental setup usually make the results not generalizable. In addition, results reported in the literature are usually quite similar, but such similitude is often related to limitations in the representativeness of the datasets, and not to the methods themselves. Thus, instead of comparing the results yielded by different approaches, a qualitative comparison is carried out, with the classes of techniques and applications shown in Table 2 as basis.

Almost all regression methods used in the context of data fusion are of the linear type. They attempt to fit a line that fits the data observed, thus providing a means for predicting values that are not present in the training datasets. In general, this type of technique can be used only when the different sources of data are of the same type or can yield features that are highly compatible. This kind of approach is still commonly used to derive soil properties, as the sensors used in this kind of application generate strings of numbers that can be easily made compatible, but it is seldom adopted in other applications.

STARFM is a data fusion method created specifically to explore the temporal and spatial characteristics of satellite images in order to generate synthetic images that combine the high temporal and spatial resolutions provided by different sensors [130]. This technique and its derivatives [122,131,132] have been successfully applied for more than 15 years, being particular prevalent for fusing Landsat and MODIS images [20,42,53,55,57,58,107,108,110,116,122,126]. Despite the growth of machine learning and deep learning techniques, the fusion of satellite imagery is still dominated by this kind of approach. There are two main reasons for this. First, these techniques were specifically designed for this type of application and have been continuously perfected, achieving high levels of accuracy and robustness. Second, although machine learning and deep learning techniques can deliver good results, they are susceptible to regularization and overfitting issues that cannot be easily avoided with satellite images [133].

Geostatistical tools, such as kriging, cokriging, and Gaussian anamorphosis, are frequently used for the delineation of homogeneous zones in agricultural areas. This is accomplished by combining different types of variables into a map according to predefined criteria. This type of approach has some competition from machine learning-based models (e.g., K-means clustering), but the latter is usually applied only when digital images are employed in combination with soil measurements [28].

PCA aims at removing redundancies that are often found in data, retaining only the components that provide new information. Although PCA has been used in a wide variety of situations and types of data, in the case of data fusion, its use has been most applied when images are combined with spectroscopy data [77,80], or as a preliminary step to prepare the data to be processed by the actual fusion models [35,41].

In the context of data fusion, Kalman filters are almost exclusively applied to combine inertial and position measurements to aid in the navigation and positioning of autonomous vehicles. This is a well-established approach that is dominating this kind of application. Although some alternative techniques have been proposed [16,98], there is not enough evidence in the literature to favor any given method over Kalman filters.

Machine learning techniques have been applied to a wide variety of problems since (at least) the 1990s. In the case of data fusion in agriculture, techniques, such as fuzzy logic [18], random forest [78,82], support vector machines [83,92,113], k-nearest neighbors [90], and shallow neural networks [24,69,85] have been employed, often showing advantages when digital images were involved, but without ever dominating other strategies. This began to change with the inception of deep neural networks. Deep learning is a branch of machine learning in which the models have deep architectures; that is, the neural networks have many layers with well-defined purposes [134]. They are particularly well suited to deal with digital images and, with the exception of satellite images, this type of technique is quickly becoming the standard for image data fusion [39]. The downside of this success is that deep learning is frequently to unsuitable datasets. Deep learning models are known to require a large number of samples to properly capture the data distributions of the classes to be considered [1]. The minimum number of samples depends on several factors, but in general, the larger the variabilities associated with the problem, the more samples are needed. This is bad news for agricultural applications because the number of factors that introduce variabilities in image datasets captured in the field is very large [1]. When the dataset lacks variability and training and test samples come from the same dataset (which is almost always the case), accuracies tend to be unrealistically high, and the trained model will almost certainly suffer from overfitting and will tend to fail when fed with new samples. Image augmentation is often used to mitigate this problem, but in many cases this process is not applied correctly, thus aggravating the overfitting problem [4]. The literature has strong evidence that deep learning is indeed the best approach when dealing with all types of digital images [21,22,39], as long some of the pitfalls associated with this type of approach are carefully avoided.

Decision rules [74], majority rules [46,117,121], and model output averaging [30] are all relatively simple techniques applied in high-level fusion with the purpose of combining the information extracted by different models. Because model selection tends to have a greater impact on the fusion effectiveness, the combination of the model outputs is usually carried out using one of these standard approaches.

### 3.5. Limitations of Current Studies

One problem that plagues most studies based on field data (not only those based on data fusion) is that the results are not realistically weighed against the limitations of the dataset used in the experiments. In particular, in the case of agriculture, it is very unlikely that a single dataset will cover all the variability that can be found in practice [1]. As emphatically put by Øvergaard et al. [89], “how well a model fits a calibration data set does not reveal any information on how good the model performance will be for other data, i.e., for real predictions”. In their work, the model was tested using data collected in a different year, under the justification that validating a model with data captured under very similar conditions to those used for training will almost invariably lead to unrealistic results, which unfortunately is usually the case. Indeed, when this approach was applied by Zhou et al. [71], the results obtained for different years were strongly disparate, thus revealing that the data used for training was not representative enough. Veum et al. [36] added that it is difficult to find the ideal sample distribution due to too much homogeneity and presence of extraneous factors. Additionally, even if the process of training, testing and validating is carried out properly, virtually all datasets used in research will have certain limitations, and these always need to be properly considered when analyzing the results. Unfortunately, with some exceptions [33,63,94,123], this is seldom the case. As a result, unrealistic claims are often found in scientific texts, hampering the progress on the subject.

### 3.6. Types of Data

In many cases, the level of useful information that can be extracted from different types of data are asymmetrical. However, it is important to note that the usefulness of certain types of data in isolation is not an indicator of how it will perform in combination with other data sources. In fact, the most important indicator for including or not a variable is its degree of complementarity with respect to the other ones [113]. In this context, variables that perform poorly in isolation can greatly improve the effectiveness of other variables if they have a high degree of complementarity [19]. In some cases, even if all variables perform poorly individually, they can yield good results when fused together [64]. Conversely, variables that perform well in isolation can match poorly with other variables if they share a high degree of redundancy. Poor synergies [86,90] and high levels of redundancy [102] between different types of data can even lead to the deterioration of the results. However, it is also important to consider that, in some contexts, even if results do not improve with the inclusion of new data types, the robustness to conditions not considered during the development of the models may increase [30]. Moreover, the complementarity of different types of data can be increased by the application of techniques capable of decreasing redundancy (e.g., PCA). In any case, determining if it is worth to include or not a certain type of data are not trivial and usually requires thorough experiments exploring different data combinations [33,36].

Different types of data can have peculiarities that need to be taken into account in order to maximize the performance of the data fusion process. For example, the amount and quality of the information contained in thermal images is highly dependent on the time of day and weather conditions at the time of collection [18,19]. Moreover, some types of data tend to be noisy and prone to outliers, especially if generated from low cost sensors [16], which can potentially introduce error instead of improving the results. In cases like these, applying noise reduction and outlier removal techniques is recommended [33,73]. A comprehensive analysis of several outlier removal techniques in the context of water management is presented by Torres et al. [93]. Despite the problems associated with spurious data, many studies simply ignore this issue, thus limiting the level of detail and quality of the resulting fused information [68].

### 3.7. Other Issues

In its most basic definition, the term “support” refers to the size or volume associated with each data value [25]. Depending on the context, however, this term may encompass other factors. For example, in the field of geostatistical research, spatial support is related not only to the area and volume associated with the data, but also includes the shape and orientation of the spatial units involved in the measurements [27]. Differences in data representativeness can be very pronounced [95], and if not properly addressed, can cause the data fusion process to fail [25,90]. As stated by Castrignanò et al. [27], “at present, the advantages of using multi-sensor data cannot yet conceal the complexity of the problem encountered in combining disparate spatial data”. These authors added that while spatial data fusion has advanced greatly in the last years, the apparent progress can be deceiving as it is often built upon unrealistic assumptions regarding support differences. Given the complexity of the problem, more rigorous validations with more realistic data and assumptions are needed to enable the development of methods with real potential for practical applications.

Overfitting is the phenomenon in which even small noisy variations in the data distribution are captured by the model, making predictions unreliable when applied to independent data [1]. This problem is particularly pervasive with small datasets with limited variability [105]. There are many techniques that can help preventing overfitting, such as data augmentation and cross-validation. Unfortunately, with a few exceptions [34,35,60,77,105], the problems of overfitting is often ignored, thus rendering the results unreliable.

Data gap filling is usually applied to satellite data to compensate for cloud cover and other factors that cause data to be lost or unusable. This can be done either by using data from other satellites or employing data collected at other levels, especially proximal, but the latter does not always improves results [51]. Data gap filling can also refer to the improvement of spatial completeness for a more detailed representation of a given area [42]. Having multiple sensors, even if they are of different types, can also help to deal with situations in which one of the data sources become unavailable for a certain amount of time due to hardware or communication problems [14]. In these cases, even if the effectiveness of the system is diminished, useful information can still be produced until the problem is resolved.

Owing to different time of acquisition, platform modes, and spectral bands of the source images, direct fusion leads to a loss of information and even the failure of fusion [68]. Currently, the most effective way to minimize this problem is by applying image registration, which has, as a main goal, to spatially match different images [19]. This step, which is often pointed out as the most important step in data fusion processes involving images [65], is essential to guarantee that the data present in different types of images are correctly fused into more complete and informative data [18]. This can be challenging, especially if the images being matched are obtained at different scales, at different bands of the spectrum, with different spatial resolutions, or are captured in areas with complex objects and topography. Additionally, the agricultural environment is highly dynamic, with plant canopies changing positions due to the wind or animals moving across the field, among other elements that may change between image captures [37]. To make matters even more complicated, significant differences between images may exist, especially if these are obtained by different acquisition methods [68]. In many cases, some kind of transformation capable of aligning the points in different images according to a given reference system is required [65].

## 4. Conclusions

Considerable effort is being made toward finding effective ways at dealing with the wealth of data that are currently being generated in the agricultural environment. Considerable progress has been made, but there are many factors that still prevent techniques that are based on data fusion from being more widely employed in practice. Three of these factors appear to be particularly relevant. First, although large amounts of data are being generated, the complexity of the agricultural environment is such that the databases being generated are not enough to cover all variabilities found in practice. As a result, models and methods proposed in the literature tend to fail under real practical conditions. Second, as the datasets used in research almost invariably have some gaps, the results reported on in scientific articles need to be weighed against those limitations. Unfortunately, this is seldom the case, and often those same results serve as the basis for the development of technologies that will likely fail. Third, even if the technology is robust enough for practical use, there are many technological, economic, political, social, and environmental barriers that prevent its adoption [135].

Despite these hurdles, the potential for growth is still there. In addition to technical advancements, there is some progress towards improving the quantity and quality of the data being collected. The practice of data sharing is steadily growing, with many research groups making datasets available under the findable, accessible, interoperable, and reusable (FAIR) principles [1,136]. When data come from a variety of sources, the representativeness of datasets tend to be much better. Additionally, “citizen science principles” [137,138]—calling for the involvement of individuals outside of the research community, in effort to build datasets—are being applied across different disciplines, with encouraging results. Once a technology truly brings benefits to potential users, adoption barriers tend to weaken. As a result of all of these efforts, the gap between academic research and practical adoption will likely continue to decrease.

## Figures and Tables

**Table 1:** List of acronyms.

Acronym	Meaning	Acronym	Meaning
AMSR-E	Advanced Microwave Scanning Radiometer	MLP	Multilayer Perceptron
	on the Earth Observing System	MLR	Multiple Linear Regression
ANN	Artificial Neural Network	MOA	Model Output Averaging
ASTER	Advanced Spaceborne Thermal Emission and	MODIS	Moderate-Resolution Imaging Spectroradiometer
	Reflection	MSDF-ET	Multi-Sensor Data Fusion Model for Actual
BK	Block Kriging		Evapotranspiration Estimation
BPNN	Backpropagation Neural Network	MSPI	Maximum Sum of Probabilities Intersections
CACAO	Consistent Adjustment of the Climatology	NB	Naïve Bayes
	to Actual Observations	NDSI	Normalized Difference Spectral Index
CHRIS	Compact High Resolution Imaging Spectrometer	NDVI	Normalized Difference Vegetation Index
CNN	Convolutional Neural Network	NIR	Near-infrared Spectroscopy
CP-ANN	Counter-Propagation Artificial Neural Networks	NMDI	Normalized Multiband Drought Index
CV	Computer Vision	OLI	Operational Land Imager
DEM	Digital Elevation Model	PCA	Principal Component Analysis
DNN	Deep Neural Network	PDI	Perpendicular Drought Index
DRF	Distributed Random Forest	PLSR	Partial Least Square Regression
ECa	Apparent Soil Electrical Conductivity	RF	Random Forest
EDXRF	Energy dispersive X-Ray Fluorescence	RFR	Random Forest Regression
EKF	Extended Kalman Filter	RGB	Red–Green–Blue
ELM	Extreme Learning Machine	RGB-D	Red–Green–Blue-Depth
EMI	Electromagnetic Induction	RK	Regression Kriging
ESTARFM	Enhanced Spatial and Temporal Adaptive	RTK	Real Time Kinematic
	Reflective Fusion Model	SADFAET	Spatiotemporal Adaptive Data Fusion
ET	Evapotranspiration		Algorithm for Evapotranspiration Mapping
FARMA	Fusion Approach for Remotely-Sensed Mapping	SAR	Synthetic Aperture Radar
	of Agriculture	SF	Sensor Fusion
GBM	Gradient Boosting Machine	SfM	Structure from Motion
GKSFM	Gaussian Kernel-Based Spatiotemporal Fusion Model	SKN	Supervised Kohonen Networks
GLM	Generalized Linear Model	SMLR	Stepwise Multiple Linear Regression
GNSS	Global navigation satellite system	SPA	Successive Projections Algorithm
HUTS	High-resolution Urban Thermal Sharpener	SPOT	Satellite Pour l’Observation de la Terre
INS	Inertial Navigation System	SRTM	Shuttle Radar Topographic Mission
IoT	Internet of Things	STARFM	Spatial and Temporal Adaptive Reflective Fusion
ISTDFA	Improved Spatial and Temporal Data Fusion		Model
	Approach	SVR	Support Vector Regression
kNN	k-Nearest Neighbors	TLS	Terrestrial Laser Scanning
LAI	Leaf Area Index	TRMM	Tropical Rainfall Measuring Mission
LPT	Laplacian Pyramid Transform	TVDI	Temperature Vegetation Dryness Index
LR	Linear Regression	UAV	Unmanned Aerial Vehicle
LSTM-NN	Long Short-Term Memory Neural Network	XGBoost	Extreme Gradient Boosting

**Table 2:** Categories adopted for the data fusion techniques and the data being fused.

No.	Classes of Data Fusion Technique	No.	Classes of Data Being Fused
1	Regression methods	1	RGB images
2	STARFM-like statistical methods	2	Multispectral images
3	Geostatistical tools	3	Hyperspectral images
4	PCA and derivatives	4	Thermal images
5	Kalman filter	5	Laser scanning
6	Machine learning	6	SAR images
7	Deep learning	7	Spectroscopy
8	Decision rules	8	Fluorescence images
9	Majority rules	9	Soil measurements
10	Model output averaging	10	Environmental/weather measurements
11	Others	11	Inertial measurements
		12	Position measurements
		13	Topographic records and elevation models
		14	Historical data
		15	Others

**Table 3:** References considered in this study–proximal scale. L, M, and H mean low-, mid-, and high-level data fusion, respectively. The numbers in the fourth column are those adopted in Table 2 for each “fused data” class.

Reference	Application	Fusion Technique	Fused Data	Mean Accuracy
[30]	Estimation of soil indices	SF (L), MOA (H)	7	0.80–0.90
[74]	Sustainable greenhouse management	Decision rules (L)	10	N/A
[73]	Human—robot interaction	LSTM-NN (L)	11	0.71–0.97
[25]	Delineation of homogeneous zones in viticulture	GAN (L), geostatistical tools (L)	2, 9	N/A
[26] ${}^{a}$	Delineation of homogeneous zones	Kriging and other geostatistical tools (L)	2, 9	N/A
[51] ${}^{a}$	Estimation of crop phenological states	Particle filter scheme (L)	2, 6, 10	0.93–0.96
[18]	Fruit detection	LPT (L) and fuzzy logic (L)	1, 4	0.80–0.95
[31] ${}^{a}$	In-field estimation of soil properties	RK (L), PLSR (L)	3, 9	>0.5
[75]	Delineation of homogeneous management zones	Kriging (L), Gaussian anamorphosis (L)	9, 15	0.66
[76]	Delineation of homogeneous management zones	Kriging (L), Gaussian anamorphosis (L)	9, 15	N/A
[27]	Delineation of homogeneous management zones	Kriging (L),Gaussian anamorphosis (L)	9, 15	N/A
[77]	Crop nutritional status determination	PCA (L)	7, 8	0.7–0.9
[22]	Detection of olive quick decline syndrome	CNN (M)	1	0.986
[65] ${}^{b}$	Monitoring Agricultural Terraces	Coregistering and information extraction (L/M)	5	N/A
[78]	Prediction of canopy water content of rice	BPNN (M), RF (M), PLSR (M)	2	0.98–1.00
[11]	Localization of a wheeled mobile robot	Dempster–Shafer (L) and Kalman filter (L)	11, 12	0.97
[19]	Immature green citrus fruit detection	Color-thermal probability algorithm (H)	1, 4	0.90–0.95
[28] ${}^{a}$	Delineation of management zones	K-means clustering (L)	2, 9, 14	N/A
[79]	Segmentation for targeted application of products	Discrete wavelets transform (M)	1	0.92
[12]	System for agricultural vehicle positioning	Kalman filter (L)	11, 12	N/A
[13]	System for agricultural vehicle positioning	Kalman filter (L)	11, 12	N/A
[67] ${}^{a}$	Yield gap attribution in maize	Empirical equations (L)	15	0.37–0.74
[32]	Soil environmental quality assessment	Analytic hierarchy process, weighted average (L)	15	N/A
[33]	Predict soil properties	PLSR (L)	7, 9, 13	0.80–0.96
[14]	System for agricultural vehicle positioning	Discrete Kalman filter (L)	11, 13	N/A
[34]	Estimating soil macronutrients	PLSR (L)	7, 9	0.70–0.95
[20]	Citrus fruit detection and localization	Daubechies wavelet transform (L)	1, 2	0.91
[15]	Estimation of agricultural equipment roll angle	Kalman filtering (L)	11	N/A
[80]	Predicting toxic elements in the soil	PLSR, PCA, and SPA (L/M)	7, 8	0.93–0.98
[68] ${}^{a}$	Review: image fusion technology in agriculture	N/A	N/A	N/A
[81]	Heterogeneous sensor data fusion	Deep multimodal encoder (L)	10	N/A
[82]	Agricultural vulnerability assessments	Binary relevance (L), RF (L), and XGBoost (L)	10,14	0.67–0.98
[35]	Prediction of multiple soil properties	SMLR (L), PLSR (L), PCA/SMLR combination (L)	7, 9	0.60–0.95
[83]	Prediction of environment variables	Sparse model (L), LR (L), SVM (L), ELM (L)	10	0.96
[64] ${}^{a}$	Estimation of biomass in grasslands	Simple quadratic combination (L)	2, 15	0.66–0.88
[23]	Plant disease detection	Kohonen self-organizing maps (M)	3, 8	0.95
[84]	Water stress detection	Least squares support vectors machine (M)	3, 8	0.99
[85]	Delineation of water holding capacity zones	ANN (L), MLR (L)	7, 9	0.94–0.97
[86]	Potential of site-specific seeding (potato)	PLSR (L)	2, 9	0.64–0.90
[87]	3D characterization of fruit trees	Pixel level mapping between the images (L)	4, 5	N/A
[88]	Measurements of sprayer boom movements	Summations of normalized measurements (L)	11	N/A
[10] ${}^{a,b}$	Review: IoT and data fusion for crop disease	N/A	N/A	N/A
[89]	Prediction of wheat yield and protein	Canonical powered partial least-squares (L)	7, 10	0.76–0.94
[69] ${}^{a}$	Wheat yield prediction	CP-ANN (L), XY-fused networks (L), SKN (L)	2, 7	0.82
[90]	Topsoil clay mapping	PLSR (L) and kNN (L)	7, 9, 13	0.94–0.96
[21]	Fruit detection	CNN (L); scoring system (H)	1, 2	0.84
[37]	3D reconstruction for agriculture phenotyping	Linear interpolation (L)	1, 10	N/A
[29]	Delineation of site-specific management zones	CoKriging (L)	2	0.55–0.77
[91]	Orchard mapping and mobile robot localization	Laser data projection onto the RGB images (L)	1, 5	0.97
[24]	Modelling crop disease severity	2 ANN architectures (L)	10, 15	0.90–0.98
[92]	Tropical soil fertility analysis	SVM (L), PLS (L), least squares modeling (L)	2, 8	0.30–0.95
[93]	Internet of things applied to agriculture	Hydra system (L/M/H)	9, 10, 15	0.93–0.99
[70] ${}^{a,b}$	Review: data fusion in agricultural systems	N/A	N/A	N/A
[36]	Soil health assessment	PLSR (L)	7, 9	0.78
[94]	Prediction of Soil Texture	SMLR (L), PLSR (L) and PCA (L)	7, 8	0.61–0.88
[95]	Rapid determination of soil class	Outer product analysis (L)	7	0.65
[16]	Navigation of autonomous vehicle	MSPI algorithm with Bayesian estimator (L)	11, 12	N/A
[38] ${}^{b}$	Detection of cotton plants	Discriminant analysis (M)	2, 7	0.97
[96]	Map-based variable-rate manure application	K-means clustering (L)	2, 9	0.60–0.93
[17]	Navigation of autonomous vehicles	Kalman filter (L)	11, 12	N/A
[97]	Robust tomato recognition for robotic harvesting	Wavelet transform (L)	1	0.93
[98]	Navigation of autonomous vehicle	Self-adaptive PCA, dynamic time warping (L)	1, 11	N/A
[99]	Recognition of wheat spikes	Gram–Schmidt fusion algorithm (L)	1, 2	0.60–0.79

^*a*^ Also explores satellite data. ^*b*^ Also explores aerial data.

**Table 4:** References considered in this study–aerial scale. L, M, and H mean low-, mid-, and high-level data fusion, respectively. The numbers in the fourth column are those adopted in Table 2 for each “fused data” class.

Reference	Application	Fusion Technique	Fused Data	Mean Accuracy
[100]	Root zone soil moisture estimation	NN (M), DRF (M), GBM (M), GLM (M)	2,11	0.90–0.95
[101]	Gramineae weed detection in rice fields	Haar wavelet transformation (L)	1, 2	0.70–0.85
[65] ${}^{a}$	Monitoring agricultural terraces	Coregistering and information extraction (L)	5	N/A
[66] ${}^{b}$	Spectral–temporal response surfaces	Bayesian data imputation (L)	2, 3	0.77–0.83
[102]	Phenotyping of soybean	PLSR (L), SVR (L), ELR (L)	1, 2, 4	0.83–0.90
[39]	Soybean yield prediction	PLSR (M), RF (M), SVR (M), 2 types of DNN (M)	1, 2, 4	0.72
[52] ${}^{b}$	Crop monitoring	PLSR (M), RF (M), SVR (M), ELR (M)	1, 2	0.60–0.93
[40] ${}^{b}$	Evapotranspiration estimation	MSDF-ET (L)	1, 2, 4	0.68–0.77
[10] ${}^{a,b}$	Review: IoT and data fusion for crop disease	N/A	N/A	N/A
[103]	Arid and semi-arid land vegetation monitoring	Decision tree (L/M)	3, 5	0.84–0.89
[41]	Biomass and leaf nitrogen content in sugarcane	PCA and linear regression (L)	2, 5	0.57
[70] ${}^{a,b}$	Review: data fusion in agricultural systems	N/A	N/A	N/A
[104]	Navigation system for UAV	EKF (L)	11, 12	0.98
[38] ${}^{a}$	Detection of cotton plants	Discriminant analysis (M)	2	0.97
[71] ${}^{b}$	Vineyard monitoring	PLSR (M), SVR (M), RFR (M), ELR (M)	2	0.98

${}^{a}$ Also explores proximal data. ${}^{b}$ Also explores satellite data.

**Table 5:** References considered in this study–orbital scale. L, M, and H mean low-, mid-, and high-level data fusion, respectively. The numbers in the fourth column are those adopted in Table 2 for each “fused data” class.

Reference	Application	Fusion Technique	Fused Data	Mean Accuracy
[42]	Soil moisture mapping	ESTARFM (L)	2	0.70–0.84
[45]	Crop type mapping	2D and 3D U-Net (L), SegNet (L), RF (L)	2, 6	0.91–0.99
[43]	Estimation of surface soil moisture	ESTARFM (L)	2	0.55–0.92
[26] ${}^{a}$	Delineation of homogeneous zones	Kriging and other geostatistical tools	2, 9	N/A
[51] ${}^{a}$	Estimation of crop phenological states	Particle filter scheme (L/M)	2, 6, 10	0.93–0.96
[53]	Evapotranspiration mapping at field scales	STARFM (L)	2	0.92–0.95
[31] ${}^{a}$	In-field estimation of soil properties	RK (L), PLSR (L)	3, 9	>0.5
[59]	Estimation of wheat grain nitrogen uptake	BK (L)	2, 3	N/A
[44]	Surface soil moisture monitoring	Linear regression analysis and Kriging (L/M)	2, 15	0.51–0.84
[46]	Crop discrimination and classification	Voting system (H)	2, 6	0.96
[9]	Review on multimodality and data fusion in RS	N/A	N/A	N/A
[47]	Crop Mapping	Pixelwise matching (H)	2, 6	0.94
[72]	Review on fusion between MODIS and Landsat	N/A	N/A	N/A
[107]	Mapping crop progress	STARFM (L)	2	0.54–0.86
[66] ${}^{b}$	Generation of spectral–-temporal response	Bayesian data imputation (L)	2, 3	0.77–0.83
[28] ${}^{a}$	Delineation of management zones	K-means clustering (L)	2, 9, 14	N/A
[114]	Mapping irrigated areas	Decision tree (L)	2	0.67–0.93
[54]	Evapotranspiration mapping	Empirical exploration of band relationships (L)	2, 4	0.20–0.97
[67] ${}^{a}$	Yield gap attribution in maize	Empirical equations (L)	15	0.37–0.74
[63]	Change detection and biomass estimation in rice	Graph-based data fusion (L)	2	0.17–0.90
[108]	Leaf area index estimation	STARFM (L)	2	0.69–0.76
[55]	Evapotranspiration estimates	STARFM (M)	2	N/A
[115]	Classification of agriculture drought	Optimal weighting of individual indices (M)	2	0.80–0.92
[56]	Mapping daily evapotranspiration	STARFM (L)	2	N/A
[20]	Mapping of cropping cycles	STARFM (L)	2	0.88–0.91
[116]	Evapotranspiration partitioning at field scales	STARFM (L)	2	N/A
[68] ${}^{a}$	Review: image fusion technology in agriculture	N/A	N/A	N/A
[52] ${}^{b}$	Crop monitoring	PLSR (M), RF (M), SVR (M), ELR (M)	1, 2, 4	0.60–0.93
[113]	Mapping of smallholder crop farming	XGBoost (L/M and H), RF (H), SVM (H), ANN (H), NB (H)	2, 6	0.96–0.98
[64] ${}^{a}$	Estimation of biomass in grasslands	Simple quadratic combination (L/M)	2, 15	0.66–0.88
[40] ${}^{b}$	Evapotranspiration estimation	MSDF-ET (L)	1, 2, 4	0.68–0.77
[117]	Semantic segmentation of land types	Majority rule (H)	2	0.99
[118]	Eucalyptus trees identification	Fuzzy information fusion (L)	2	0.98
[10] ${}^{a,b}$	Review: IoT and data fusion for crop disease	N/A	N/A	N/A
[69] ${}^{a}$	Wheat yield prediction	CP-ANN (M), XY-fused networks (M), SKN (M)	2, 7	0.82
[112]	Drought monitoring	RF (M)	2, 15	0.29–0.77
[48]	Crop type classification and mapping	RF (L)	2, 6, 13	0.37–0.94
[119]	Time series data fusion	Environmental data acquisition module	10	N/A
[57]	Evapotranspiration prediction in vineyard	STARFM (L)	2	0.77–0.81
[109]	Daily NDVI product at a 30-m spatial resolution	GKSFM (M)	2	0.88
[49]	Crop classification	Committee of MLPs (L)	2, 6	0.65–0.99
[6]	Multisource classification of remotely sensed data	Bayesian formulation (L)	2, 6	0.74
[111]	Fractional vegetation cover estimation	Data fusion and vegetation growth models (L)	2	0.83–0.95
[120]	Land cover monitoring	FARMA (L)	2, 6	N/A
[121]	Crop ensemble classification	mosaicking (L), classifier majority voting (H)	2	0.82–0.85
[70] ${}^{a,b}$	Review: data fusion in agricultural systems	N/A	N/A	N/A
[50]	In-season mapping of crop type	Classification tree (M)	2	0.93–0.99
[122]	Building frequent landsat-like imagery	STARFM (L)	2	0.63–0.99
[58]	Evapotranspiration mapping	SADFAET (M)	2	N/A
[123]	Temporal land use mapping	Dynamic decision tree (M)	2	0.86–0.96
[124]	High-resolution leaf area index estimation	STDFA (L)	2	0.98
[125]	Monitoring cotton root rot	ISTDFA (M)	2	0.79–0.97
[110]	Monitoring crop water content	Modified STARFM (L)	2	0.44–0.85
[105]	Soil moisture content estimation	Vector concatenation, followed by ANN (M)	2, 6	0.39–0.93
[126]	Impact of tile drainage on evapotranspiration	STARFM (L)	2	0.23–0.91
[127]	Estimation of leaf area index	CACAO method (L)	2	0.88
[106]	Mapping winter wheat in urban region	SVM (M), RF (M)	2, 6	0.98
[128]	Leaf area index estimation	ESTARFM (L), linear regression model (M)	2	0.37–0.95
[71] ${}^{b}$	Vineyard monitoring	PLSR (M), SVR (M), RFR (M), ELR (M)	2	0.98

^*a*^ Also explores proximal data. ^*b*^ Also explores aerial data.

## Data Availability

Not applicable.
